# Clinical Society of the New York Polyclinic Medical School and Hospital

**Published:** 1905-02

**Authors:** 


### SOCIETY REPORTS.

### CLINICAL SOCIETY OF THE NEW YORK POLYCLINIC MEDICAL SCHOOL AND HOSPITAL.

Stated meeting, held January 9, 1905, the President, Dr. Daniel S. Dougherty, in the chair.

### PATIENT WITH TUBERCULOSIS OF WRIST.

Dr. V. C. Pedersen showed this patient. Several years ago she went to one of the large hospitals of this city with a condition presenting the early stages of arthritis of the wrist. Expectant treatment was adopted and was followed by swelling of both the hand and forearm. Notwithstanding the fact that the metacarpal phalangeal joints were ankylosed, no effort was made to reduce the ankylosis by the hospital staff. The patient was then recommended to go to the central part of the State, where she improved in general health There the supposed diagnosis of tuberculous synovitis of the extensor tendon was made and explored operatively. Six months later she presented herself to the speaker, who did a resection of the wrist. The trapezium and the synovial membrane between it and the thumb were in fairly healthy condition and were not removed; likewise, the membrane between the radius and the ulna, and the pouches between the bases of the fourth inner metacarpal bones. About a year later pain and swelling began to appear on the outer side of the hand, and a secondary operation was performed, when it was found that the trapezium and the synovial membrane were tuberculous. These were removed. The wrist was opened a year after that, and extensive tuberculosis of the metacarpal surfaces of the ulna and radius and likewise of the other bones was found, and the third operation was almost as extensive as the original resection. The arm was elongated, allowing the cavity which was left to fill, with blood-clot, which organized without suppuration. This was done at the suggestion of Dr. Dawbarn, who was called in as consultant, but personally, the speaker thought it much better to accept shortening and approximate the bones of the metacarpus to the bones of the forearm. Cosmetically, the condition of the patient's wrist is very satisfactory, and it is hoped that all tuberculous foci have been removed, but it is only five weeks after the operation and too soon to tell the final result.

Dr. A. Lyle said that for some years it has been his custom to adopt the expectant treatment in cases of tuberculosis of the wrist, but to-day he was in favor of operating at once.

Dr. J. A. Bodine said that in tuberculosis of the hip-joint where it was possible to get more or less perfect rest, there are reasons for adopting the expectant method of treatment; but, he thought, in the wrist, where the anatomical configurations of the joint are so complex, and where it is so impossible to get physiological rest, it was better to operate at once. He thought the result in the case shown very satisfactory, but it was entirely too soon to judge of the outcome of the operation.

PATIENT WITH ARTHRITIS OF KNEE—PROBABLY GONORRHEAL.

Dr. Pedersen also presented a patient, the victim of a druggist's error, who, realizing himself to be in the early stages of gonorrhea, entered a drugstore and asked for flexible medicated urethral bougies, stating to the druggist the purpose for which they were intended. Instead of bougies, the druggist dispensed pure silver nitrate caustic in sticks, which the patient, in ignorance, inserted with the following results: Excruciating pain and burning, after a few moments followed by total absence of feeling; later, tremendous swelling and inflammation of the part appeared; total inability to urinate supervened, so that the use of the catheter was absolutely necessary. The fifth day after the accident there were silver nitrate burns on both thighs as far as the knees, two or three of them having penetrated almost or quite through the skin, as subsequent scarring proved. The entire skin sheath of the penis was swollen to several times its normal thickness; the margin of the foreskin was burned raw almost completely around; the glans was violently inflamed, swollen and edematous. It was impossible to retract the foreskin even partially. The meatus was with difficulty brought into view, and was completely filled with the typical whitish slough of silver nitrate burn, which extended backward fully four inches and gave the urethra the feeling of boggy rotten rubber tubing. Behind the slough the urethra was distinctly tender. The prostate was not investigated. The urethra was cocainized, a soft Lisle thread catheter was inserted into the bladder and about fourteen ounces of clear urine were withdrawn. The patient was sent to the hospital and lead and opium wash applied externally. The copious discharge was found to contain gonococci. The patient was catheterized once in twelve hours in order to decrease the likelihood of infecting the bladder. Within twenty-four hours after admission drainage was carried out through the perineum with the double purpose of stopping the catheterizing and of putting the urethra at rest. At the time of the operation the slough came away in mass and the urethra and bladder were irrigated thoroughly and copiously with very hot potassium permanganate solution 1–4000. Subsequent treatment was irrigation of the bladder with hot potassium permanganate solution and hot boric acid water two or three times a day. The same substance were also used on the urethra. At the time of operation 32 F. sound was passed without force through the urethra. About six days later 25 and 27 F. straight sounds were passed with some pain to the bulb of the urethra. Later, under gas anesthesia 30 F. straight sound was passed. All subsequent examinations of the discharge for gonococci have been negative, and at the present time the patient passes, with the aid of cocainization, 29 F. sound quite easily.

On the day following the original operation the foreskin was slit dorsally from end to end, in order to be sure that there were no severe burns of the glans. Circumcision will be carried out upon the patient within a few weeks. Whether it will be necessary to do an internal urethrotomy through the scar of the deepest burn, in order to gain space, remains to be seen.

### HYSTERECTOMY SPECIMEN WITH HISTORY.

Dr. Pedersen also presented a specimen which had been removed from a patient on whom hysterectomy for extensive laceration had been performed. The patient's history previous to marriage was negative. Ten months after marriage she was delivered of a full term dead child, with instruments. Puerperium lasted ten weeks with severe symptoms. Two miscarriages the following year were followed by pregnancy some months later, with what was considered menstrual flow during the first six months. She was delivered of a full term male child by the breach with little trouble, puerperium lasting ten days. This child is strong and healthy at the present time. Eighteen months later she was again pregnant, giving birth to a second male child. The mother nearly lost her life through hemorrhage. This boy is also living and in good health at the present time. Nursing was impossible through weakness of the mother; she was, however, able to nurse her first son for fifteen months. In the summer of the following year she was again pregnant, and during the winter nearly had a miscarriage. She gave birth, however, to a female child. This labor was nearly natural, and no physician was on hand at the time. This child is also living and in good health. A year later she was taken sick with apparently accidental hemorrhages during a pregnancy. Upon her physician's advice she went to a hospital in Connecticut, where a curetting was carried out, four days after which she had a miscarriage of apparently a six weeks' fetus.

Later in the same year she again went to the same institution, where she was treated for syphilis. Her doctor, doubting the diagnosis, sent her to a specialist in this city, who positively denied the existence of syphilis in the eruption. In December of the same year the patient had a miscarriage. In December, 1903, she was sent to a New York hospital for the repair of a very extensive laceration extending almost to the left horn of the uterus. Suture of the tear was done, and secondary hemorrhage almost carried the patient off. After her recovery she was told to return in October for examination, when she was informed that hysterectomy was necessary, but her condition would hardly warrant the operation. Dr. Pedersen saw her late in October in excellent general health and spirits and showing a uterus normal in size, form and consistency, freely movable from the back, right side and front, but distinctly adherent on the left, where an extensive infiltration was present associated with an extensive tear, the depth of which was not fully explored. Hysterectomy was advised and performed November 17, 1903. The patient made an uneventful recovery and was discharged December 15th. The operation which proved somewhat difficult was carried out through an abdominal incision.

When sent to New York the patient had been suffering intolerably for two or three months at each menstruation and had been scarcely free of pain at any time. Since the hysterectomy the doctors say she has been entirely free of pain, well and happy, recognizing each menstrual epoch, and has had a number of hemoptyses, which subsequently changed to epistaxis at the time considered by her to represent her monthly flow. A report from the doctors state that she has gained in weight and her monthly flow is still recognizable, being accompanied by epistaxis frequently, occasionally by hemoptysis, and once by hemorrhage from the bowels and by the usual nervous disturbances.

Dr. J. Riddle Goffe said that the history of the patient upon whom the hysterectomy had been performed, as well as the ultimate outcome, justified Dr. Pedersen's procedure. The speaker was surprised that the patient suffered with the symptoms of vicarious menstruation, and should have nose bleed at the regular monthly recurrences. He said that examination has proved that the fallopian tubes play a very active part in the shedding of blood, and the speaker had operated on two cases in which he had had the opportunity of watching the dripping of blood from the uterine ends of the tubes into the vagina.

RESULTS OF TREATMENT OF ENDOCARDITIS IN CHILDREN.

Dr. Lucy Jones showed several children whom she had treated for endocarditis. The first patient showed, a boy about eight years, was first seen by the speaker in July, 1902. His mother then said that he had for three days suffered from a fever for which she could not account. As is her custom, the speaker stripped him and began examination from the head. His tonsils were swollen, lungs negative, heart presented a presystolic murmur at the apex, which accounted for his fever. He was put to bed, and absolute rest insisted on; salicylate of soda given, an ice-bag put over his heart, and he was given a milk and cereal diet. Temperature gradually dropped each day until normal at the end of a week, but the pulse kept up, so the ice-bag was kept on for three weeks. At the end of the fourth week he began to sit up a little each day. When he first sat up the murmur was very faint, but after a few days, appeared very markedly. At present he walks and plays without any inconvenience to himself, and no murmur is apparent until he walks rapidly up and down the room a few times, when it becomes very marked.

The second patient, a girl about six years, came to the dispensary with a history of three previous attacks of rheumatism. At this time she had been sick for three days, with pain and swelling in the ankles, high fever, which would not yield to treatment. Examination of the heart revealed a systolic and presystolic murmur at the apex. She was also put to bed with an ice-bag over her heart, and one week later, had difficulty in breathing, was pale and anemic, and examination showed that the cardiac dulness extended from the articulation of the costal cartilage on the right side to one inch beyond the nipple on the left side. The heart sounds were distant and muffled. The diagnosis was pericarditis, with effusion. A fly-blister was put over the heart, and the effusion gradually disappeared, and the patient developed the friction rub of pericarditis, which also disappeared. At the end of a month the child was allowed to sit up. She runs and plays without difficulty, has not had an attack since, and is now perfectly well. No murmur is heard.

The next patient shown had been under treatment for three years. He gave no history of rheumatism. When first seen, he shook all over and could not control his muscles nor hold anything in his hand. There was an aortic systolic murmur with slight rise of temperature. He was put to bed, an ice-bag applied, and salicylate of soda administered, and within three days he had a presystolic murmur at the apex, as well as the aortic. In two weeks the chorea disappeared, but the heart was the same. At the end of one month he had an aortic, systolic, presystolic and systolic murmur at apex; at the end of three months, he had a systolic murmur at the apex. When he was allowed to get up, two months later, he had an attack of indigestion, and three days later the heart was attacked a second time, which lasted six weeks, when he was up again for two months, and then in bed again for five. He had hypertrophy of the left side of the heart and acute dilatation of the right side, with attacks of angina. He exercises mildly, takes digitalis in small doses, which are increased when the heart does not do its work well, as the right side dilatation is still present.

The fourth patient had an attack of rheumatism about a year ago with endocarditis, which was entirely cured, but later had two slight attacks of rheumatism, for which her mother treated her at home. She was allowed to run around the house, and tonight she had a presystolic and a systolic heart murmur, the result of not having been treated during the last attack of rheumatism.

In the speaker's opinion, the rest was the most important part of the treatment, the ice-bag, salicylate of soda, etc., being but secondary considerations.

Dr. F. Beal opened the discussion, and said that he thought the key-note of the whole treatment was in one word, "rest."

Dr. M. Packard said that the treatment of endocarditis in children was much more promising than in adults, and if the disease was recognized early enough, even if ulcerous, there was hope of a cure. In the first case shown, he thought that the heart was not compensated because the murmur, although absent at other times, was brought out by exercise. Compensation in these cases can often be brought about by stimulation, and the best method is by judicious diet. He advised feeding the children six or eight times a day, and very little at each feeding, and not allowing them to drink much liquid at any one time, as rapid filling of the vessels of the heart would cause dilatation. He thought that puberty often brought on compensation, as then the muscles both hypertrophied and dilated as well.

Dr. Jones said that the boy was now on a diet, his exercises were watched, and at the first sign of fever his mother put him to bed and kept him there until entirely well.

SPECIMEN OF PRIMARY EPITHELIOMA OF TONSIL.

Dr. Robertson presented this specimen, which he had removed from the patient a month previously. The history was that nine months ago a slight swelling appeared at the angle of the right side of the jaw which gradually increased in size, but there was no sore throat. Examination revealed a much ulcerated tonsil. Epithelioma was suspected and four days later the speaker removed part of the tonsil and eight or ten glands and had them examined, and the diagnosis of primary epithelioma of the tonsil was made. The enlargement of his throat at the present time has developed during the last four weeks. X-rays are being used and boric acid and aristol are being blown in as a powder on the tonsil growth.

Dr. Bodine said that in all cases of suspected malignancy of the tonsil, physiological doses of iodide should be given and the dose should be increased until one of two things happen: either the disappearance of the lesion or symptoms of constitutional iodism, and he had, in his own practice, administered as much as 900 grains iodides in one day with resulting disappearance of the lesion. As to the removal of a piece of the tonsil for microscopical examination, the wisdom of such procedure is open to question, unless the patient is fully prepared for immediate operation upon the report of malignancy. The clinical diagnosis of malignancy in the case shown is sufficient without the microscope, and in the event of a different report by the pathologist, he would place his clinical diagnosis above that of the microscope and operate anyhow.

Dr. Milton Franklin said that the microscopical picture presented by this condition could not always be accepted as final. He recalled a patient who had received a physiological dose of iodides without effect and examination of a section of the tonsil under the microscope presented all the structural characteristics of epithelioma; two weeks later a second section was removed which showed no evidence of epitheliomatous infection; a third section two weeks later presented the typical picture of epithelioma, as did the clinical aspect of the case, which resembles multiple epithelioma of the side of the face, tonsil and lip. Potassium iodide was administered and when the iodide was removed the lesion disappeared entirely. X-ray treatment, he thought, helped those cases more or less, as the lesions grew smaller and often disappeared, but ultimately the condition again developed and the outcome was rarely satisfactory.

Dr. Dougherty said that he had seen two cases, both of whom were treated with iodides and both of whom died before the physiological dose was reached. He had tried to persuade both patients to submit to early and radical operation, but without success. In one case the growth was very gradual for twelve or fourteen months, and then suddenly developed very rapidly.

Dr. R. C. Myles said that in his opinion the only hope for cure in these cases lay in early and radical operation. He had at the present time a patient suffering with epithelioma of the tonsil who absolutely refused to be operated on and who was being treated with X-ray but, although the mass was becoming smaller, the speaker had no confidence in the ultimate result.

Dr. Robertson in closing the discussion, said that his patient had been given potasum iodide from 5 to 10 grains, increased daily in saturate solution. The advisibility of a secondary operation was discussed but was not deemed wise because of the almost certainty of recurrence in all of these cases.

The paper of the evening was read by Dr. Joseph Y. Mangum and was entitled:

### THE ABDOMINAL VS. THE VAGINAL ROUTE IN GYNECOLOGICAL OPERATIONS.

He said, in part, that whether to approach a pelvic lesion by the abdominal or the pelvic route was a question often difficult to answer. It can only be answered correctly after a careful examination of the history, and especially the physical conditions has given the surgeon a clear idea of the intra-pelvic conditions and of what he must accomplish to secure the greatest possible permanent benefit.

The choice of a route is never difficult in cases of recent extensive bilateral pelvic suppuration, pelvic abscess, puerperal sepsis, or in any collection of pus, blood or other fluid low down in the posterior cul-de-sac. Here the way through the vagina should usually be chosen, because by this route we can evacuate the septic material, drain and cure, with the least possible danger of infecting the general peritoneal cavity. You have by this route the advantages of a small wound, a small raw area to absorb the septic material, and a natural sewer to drain through, avoidance of exposure in handling the intestines, and consequently less immediate risk and shock.

The vaginal route is, for a large proportion of cases, impracticable, and especially for the man of limited experience. The long, narrow vagina, the small vagina, or small vagina from senile atrophy may render the field almost inaccessible. Enlarged uteri, with short, thick, broad ligaments and enlarged appendages, and with adhesions extending beyond the reach of the finger, may cause it to be impossible to complete the operation satisfactorily. Under these circumstances the abdominal route is much to be preferred and much safer.

The abdominal route is also preferred in abdominal cyst, cystic ovaries, fibroids, myoma, tubal pregnancies, general conservative work on the appendages, such as occluded tubes, adherent ovaries and tubes, hyprosalpinx, broad ligaments, cysts and displacements of the uterus. First, there is a larger field for operation, which is exposed, and the operator is not compelled to rely entirely upon the sense of touch. Diagnosis of unsuspected pelvic and abdominal lesions and complications is much easier, and pathological conditions are revealed that would pass entirely unnoticed if the operation had been performed by the vaginal route. The field of operation is much cleaner as the surgeon does not operate through one sewer of the body and between two other sewers, and one that it is impossible to sterilize. The chances of adhesions are greater, especially when gauze packings or instruments are left in the vaginal incision. Also, there is no danger of secondary infection, as in the vaginal route, and if the uterus, bladder or intestines are wounded, they can be repaired at once and satisfactorily. Also, the abdominal route presents more light for conservative work on the appendages, and there is almost always a sensitive scar after vaginal incision, and not after abdominal.

Dr. C. S. Child, Jr., opened the discussion of Dr. Mangum's paper. He said that in his opinion no route was advisable for all cases, but he thought that the vaginal route should be used much oftener than it was by the average gynecologist, though not as often perhaps, as by the vaginal operator, and had a much greater field of usefulness than the writer of the paper allowed. The operator who drains a pus-pocket through the posterior vaginal fornix is only following out the way pointed out by nature. It is a very simple procedure, but celiotomy through the anterior vaginal fornix discloses the whole pelvic field for treatment, and by this method of approach any abnormal pathological condition limited to the pelvis can be satisfactorily treated. As to the writer's statement that sensitive scars persisted after the vaginal incision more frequently than after the abdominal, it has been the speaker's experience that the opposite was the case, there being a far greater nerve distribution in the site of incision in the abdominal wall than in that of the vagina. A tense sensitive condition of the utero-sacro ligaments frequently persists for some time after a posterior incision for pus drainage, and very probably this condition is what the writer considered sensitive scar.

Dr. J. H. Burtenshaw said it was preposterous to lay down a hard and fast rule as to operations by the abdominal or vaginal route. To a certain extent each and every abnormal pelvic condition requiring surgical intervention is a case unto itself, and the character of the lesion and the depth of the pelvism, the size of the vagina, the skill of the individual operator are all factors which must be duly considered. He deprecated the teaching that one method of attack should be followed to the exclusion of the other. While it is well known that vaginal section is far less productive of shock, in the majority of instances, it by no means follows that it should be adopted in all cases.

Dr. B. Torrens said that the lesions best treated by the vaginal route were those which lie below the brim of the pelvis, and which are the product of infections which have found their entrance through the vagina. In this list should be included not only puerperal infections and free pus in the pelvis, but also pyosalpinx, occluded and adherent tubes, cysts of ovary and broad ligament, adherent and retroverted uterus and also early ectopic gestations. The advantages of the vaginal route in the above classes of cases are that in entering the peritoneal cavity, but two anatomical layers are cut, which reunite without suturing, the lesions lie between your opening and the intestines, which are less liable to injury and infection, also absence of hemostasis and ligatures; perfect drainage; shorter operation; less profound and shorter narcosis; no subsequent hernias; lower mortality.

Dr. B. H. Wells believed that in many instances the choice of route was a matter of individual skill or preference. Personally, he found the abdominal incision, in operations for the relief of uterine displacement, conservative work on the uterus or appendages, extra-uterine gestation, most cases where hysterectomy was required, and where long tumors had to be removed, with extensive or recent pelvic suppuration, the vaginal method was the best.

Dr. J. C. Taylor said that over fifty per cent. of gynecological operations were performed for pus. Now, Dr. Mangum said, that with free pus in the pelvis and free fluid in the pelvis, he advised vaginal method; but cases of fibroids, tubal pregnancies, pyosalpinx, ovarian cysts, etc., should be done through the abdomen. The hospital records of St. Luke's, Woman's and Roosevelt Hospitals for the past five years show that the pus operations done through the abdomen show a fatality of ten per cent. The operations done by the late Dr. Pryor and Dr. Cleveland through the vagina show a mortality of less than one per cent. If patients operated on through the abdomen do get well, there will be either adhesions of the omentum and intestines or the risk of a hernia in many cases. If a tubal pregnancy, for instance, ruptures before the sixth week, it will be found in the pelvic cavity, and should be opened there.

Dr. Goffe said that he found the field of the vaginal operator being gradually extended. Dr. Noble, of Philadelphia, a few years ago, operated on all pus tubes through the abdomen, with a mortality of about twenty per cent., and now attacks them through the vagina with a mortality of less than one per cent. Nothing was heard about sensitive vaginal scar until the opposition arose to the vaginal route for minor pelvic surgery. Hysterectomies had been done for years and no objection made to the operation on account of sensitive scar, and the scar from that operation is much more extensive than that arising from an anterior or posterior vaginal section for dealing with the appendages. As a matter of fact, the speaker had never met with a sensitive scar in any of his cases after vaginal section, and had probably examined as many as any one.

